# Sporadic type VI secretion in seventh pandemic *Vibrio cholerae*

**DOI:** 10.1099/mic.0.001329

**Published:** 2023-05-03

**Authors:** Alexis Proutière, Natália C. Drebes Dörr, Loriane Bader, Sandrine Stutzmann, Lisa C. Metzger, Sandrine Isaac, Nicolas Chiaruttini, Melanie Blokesch

**Affiliations:** ^1^​ Laboratory of Molecular Microbiology, Global Health Institute, School of Life Sciences, Ecole Polytechnique Fédérale de Lausanne (EPFL), CH-1015 Lausanne, Switzerland; ^2^​ Bioimaging and Optics Platform (PT-BIOP), School of Life Sciences, Ecole Polytechnique Fédérale de Lausanne (EPFL), CH-1015 Lausanne, Switzerland

**Keywords:** *Vibrio cholerae*, type VI secretion system, pandemic clade, subpopulation, bacterial heterogeneity

## Abstract

*
Vibrio cholerae* is a pathogen that causes disease in millions of people every year by colonizing the small intestine and then secreting the potent cholera toxin. How the pathogen overcomes the colonization barrier created by the host’s natural microbiota is, however, still not well understood. In this context, the type VI secretion system (T6SS) has gained considerable attention given its ability to mediate interbacterial killing. Interestingly, and in contrast to non-pandemic or environmental *V. cholerae* isolates, strains that are causing the ongoing cholera pandemic (7PET clade) are considered T6SS-silent under laboratory conditions. Since this idea was recently challenged, we performed a comparative *in vitro* study on T6SS activity using diverse strains or regulatory mutants. We show that modest T6SS activity is detectable in most of the tested strains under interbacterial competition conditions. The system’s activity was also observed through immunodetection of the T6SS tube protein Hcp in culture supernatants, a phenotype that can be masked by the strains’ haemagglutinin/protease. We further investigated the low T6SS activity within the bacterial populations by imaging 7PET *V. cholerae* at the single-cell level. The micrographs showed the production of the machinery in only a small fraction of cells within the population. This sporadic T6SS production was higher at 30 °C than at 37 °C and occurred independently of the known regulators TfoX and TfoY but was dependent on the VxrAB two-component system. Overall, our work provides new insight into the heterogeneity of T6SS production in populations of 7PET *V. cholerae* strains *in vitro* and provides a possible explanation of the system’s low activity in bulk measurements.

## Introduction

*Vibrio cholerae* is most prominently known as the causative agent of the deadly diarrhoeal disease cholera [1, 2]. However, only a minority of strains within this species carry the primary virulence factor-encoding genes, namely those encoding cholera toxin and the toxin-coregulated pilus TCP [3, 4]. Moreover, among these ‘toxigenic’ strains, only those belonging to serogroups O1 and O139 have caused pandemics in the past, while selected non-O1/non-O139 strains (e.g. the O37 serogroup [5, 6]) have been responsible for more localized outbreaks. Although most toxigenic isolates are derived from patients, the species’ primary niche is the aquatic environment [7, 8]. In this habitat, *V. cholerae* is often found in biofilms attached to chitinous exoskeletons of zooplankton [8].

During host colonization and in its natural aquatic environment, *V. cholerae* faces strong competition from other bacteria. Under such competitive conditions, bacteria often employ molecular weapons for interbacterial antagonism, such as the type VI secretion system (T6SS) [9, 10]. The T6SS is a multiprotein complex that allows the injection of toxic effector proteins into neighbouring cells [11], while sister cells are protected through the production of cognate immunity proteins [12–16]. For *V. cholerae *, both environmental and toxigenic strains carry highly similar structural gene clusters that are necessary for T6SS production [i.e. one large cluster encoding the main structural components of the T6SS machinery plus one VgrG protein and effector-immunity (E/I) pair, and two core auxiliary clusters encoding Hcp proteins as well as additional VgrG and E/I pairs] [16, 17], supporting the idea that the machinery could be beneficial in the aquatic environment and in the gut. Surprisingly, T6SS production is differentially regulated among diverse *V. cholerae* strains. Indeed, environmental and toxigenic non-pandemic strains constitutively produce their T6SS under standard laboratory conditions [18–22], explaining the preferential use of these strains in studies that address the mechanistic aspect of this secretion system [15, 23]. Strains belonging to the seventh pandemic El Tor clade (7PET), which are responsible for the ongoing cholera pandemic, however, have been described to be T6SS-silent when grown in the laboratory [14, 18, 22, 24].

Interestingly, in strains belonging to the sixth pandemic clade of *V. cholerae* (so-called classical strains), the T6SS is non-functional due to accumulated mutations in T6SS genes [25]. This raises the question of why the same phenomenon does not occur in 7PET strains. While there is evidence that it might play a role in the colonization of rodent or zebrafish hosts [26, 27], we hypothesize that the primary role of the T6SS of 7PET strains might be linked to *V. cholerae *’s environmental lifestyle. Indeed, during colonization of chitinous surfaces, the T6SS is induced by the regulatory protein TfoX [24, 28]. Another regulator, TfoY, also induces the T6SS in diverse *Vibrio* species [29, 30]. Notably, in *V. cholerae* TfoY production is regulated (post-transcriptionally) by the second messenger c-di-GMP [28, 29, 31, 32], yet the exact condition under which TfoY is produced remains unknown.

Regulation of the T6SS by the TfoX protein is niche specific, as it exclusively occurs on chitinous surfaces [24]. TfoX-mediated T6SS production, concomitantly with natural competence for transformation, also requires input from the bacterial quorum sensing (QS) circuitry [24]. In brief, both TfoX and the master regulator of QS (HapR) promote the production of the transcription factor QstR [33], which is required and sufficient to induce T6SS expression in 7PET *V. cholerae* [24, 34]. The coupling of T6SS-mediated interbacterial killing and competence in *V. cholerae* therefore results in the killing of non-kin neighbouring bacteria followed by the absorption of prey-released DNA [24, 35].

While TfoY- and TfoX-mediated induction of T6SS expression in 7PET strains is well supported by the literature, constitutive T6SS production in non-pandemic strains remains understudied. In this context, we recently reported the experimental identification of a single-nucleotide polymorphism (SNP) located in an intergenic region within the primary T6SS cluster of *V. cholerae* [36], which was concomitantly also identified by others using a bioinformatic approach [37]. We demonstrated that the exchange of this SNP (e.g. SNP conversion) was sufficient to render 7PET strains T6SS-active under laboratory conditions, while the opposite exchange silenced T6SS expression in non-pandemic isolates [36].

Upon its discovery, Pukatzki and colleagues had already demonstrated that the T6SS was silenced under laboratory conditions in 7PET *V. cholerae* strains [18], which was later confirmed given that those strains were unable to kill bacterial competitors and showed undetectable T6SS gene expression [14]. Our previous work supported these observations, as no statistically significant interbacterial competition could be observed for the tested 7PET strains under standard laboratory conditions [22, 24, 28, 36]. However, other studies suggested that the T6SS of 7PET strains might nonetheless be active upon growth in rich medium. For instance, Ishikawa *et al.* demonstrated T6SS-mediated killing of *Escherichia coli* prey by a wild-type (WT) 7PET strain, a phenotype that was enhanced by high-salinity conditions and lowered temperatures; however, those conditions or the deletion of the putative T6SS repressor gene *oscR* only marginally increased T6SS gene expression (<2-fold upregulated for most tested T6SS genes) [38]. Likewise, Cheng *et al.* reported an ~1 log reduction in *E. coli* prey recovery after competition with a 7PET strain compared to that with its T6SS-deficient derivative (under high-salinity conditions). This killing activity was reduced in a mutant that lacked the response regulator of the two-component system (TCS) VxrAB (for *Vibrio* type six secretion regulator [39]). The VxrAB TCS also plays an important role in cell wall homeostasis and regulation of iron acquisition [40, 41], and is essential for robust biofilm formation and matrix-dependent protection against T6SS insults [42, 43]. The absence of the residual gene products of the *vxr* operon (e.g. VxrC, D, E) resulted in a changed expression pattern of certain matrix genes, yet only the ∆*vxrC* strain produced biofilm structures that differed from the one formed by the WT [42].

In this study, we sought to determine why residual yet modest T6SS activity in 7PET strains remained undetected under laboratory conditions in several studies published by different research groups [14, 20, 24, 28, 36, 44]. The primary purpose of this work was therefore to understand the discrepancy between reported T6SS activity/inactivity of those strains and to develop a defined protocol that allows the investigation of the strains’ low T6SS activity *in vitro*. Briefly, we compared growth conditions and diverse 7PET predator and *E. coli* or *V. cholerae* prey strain combinations and applied various readouts to test whether T6SS activity was detectable in 7PET strains. Interestingly, by imaging *V. cholerae* at the single-cell level, we demonstrate that a small subfraction of the 7PET population was T6SS-active under standard laboratory conditions while the majority of the population did not assemble T6SS structures under the tested conditions. Moreover, we showed that this sporadic T6SS activation was temperature- and VxrAB-dependent and enhanced in the absence of type VI secretion system regulator A (TsrA; 44). Notably, this non-uniform T6SS activity within the population resulted in variable and often statistically non-significant batch culture-based results, as we demonstrate for the enumeration of interbacterial killing events or the secretion of the T6SS inner tube protein Hcp.

## Methods

### Bacterial strains and growth conditions

The bacterial strains used in this study are listed in Table 1. *V. cholerae* and *E. coli* strains were grown aerobically either in lysogeny broth (LB; 10 g l^–1^ tryptone, 5 gl^–1^ yeast extract, 10 gl^–1^ NaCl; Carl Roth) or on LB agar plates at 30 or 37 °C unless otherwise stated. Half-concentration defined artificial seawater (0.5×DASW) medium containing HEPES (Sigma-Aldrich) and vitamins [45], or 0.5×HW Marine Mix (Wiegandt) [36] was used for growth on chitin to obtain genetically engineered strains by natural transformation [46–48]. After bi- or triparental mating with *E. coli* strains, thiosulfate citrate bile salt sucrose (TCBS; Sigma-Aldrich) agar was used to counterselect *E. coli *. Counterselection based on *sacB* was performed on NaCl-free medium containing 10 % sucrose after biparental mating. When needed, arabinose (for the expression of *tfoX*, *tfoY* and *vxrB* under control of the *P*_BAD_ promoter) or antibiotics were added to the growth medium at the following concentrations: l-arabinose (0.2 or 0.02 %), kanamycin (75 µg ml^−1^), streptomycin (100 µg ml^−1^), gentamicin (50 µg ml^−1^) and ampicillin (100 µg ml^−1^).

**Table 1.** *
Vibrio cholerae* and *Escherichia coli* strains and plasmids used in this study

Strain name	Genotype/description*	Internal strain no.	Reference
*** V. cholerae ***			
A1552	Wild-type, O1 El Tor Inaba, isolated in 1991 and linked to Peruvian cholera outbreak; Rif^R^	MB_1	[71, 72]
A1552-Tn*tfoX-strep* (TnTfoX)	A1552 containing mini-Tn7-*araC*-P_BAD_-*tfoX-strep*; Rif^R^, Gent^R^	MB_3420	[28]
A1552-Tn*tfoY-strep* (TnTfoY)	A1552 containing mini-Tn7-*araC*-P_BAD_-*tfoY-strep*; Rif^R^, Gent^R^	MB_2978	[28]
A1552Δ*hapA*	A1552 with *hapA* (*VCA0865*) deleted; Rif^R^	MB_4	[73]
A1552-chg[−10box]	A1552 with site-directed mutation (AA to GC) in −10 element in intergenic region between *VCA0106* and *VCA0107* (*vipA*); Rif^R^	MB_9952	This study
A1552Δ4EI-Kan	A1552 with genes encoding the four T6SS effector/immunity pairs deleted (=Δ4EI: *ΔVCA0123-24*::*FRTΔVCA0020-21*::*FRTΔVC1418-VC1419*::FRT∆*VCA0285-86*::FRT), and with *aph* cassette inserted into ∆*VC1807;* Rif^R^, Kan^R^	MB_9525	This study
A1552Δ*vxrA*	A1552 with *vxrA* (*VCA0565*) deleted (A1552Δ*vxrA*::FRT); TransFLP; Rif^R^	MB_3806	This study
A1552Δ*vxrA* ∆*vasK*-NEW	A1552Δ*vxrA* with *vasK* (*VCA0120;* entire ORF) deleted; Rif^R^	MB_9953	This study
A1552Δ*vxrB*	A1552 with *vxrB* (*VCA0566*) deleted (A1552Δ*vxrB*::FRT); TransFLP; Rif^R^	MB_3807	This study
A1552Δ*vxrB* ∆*vasK*-NEW	A1552Δ*vxrB* with *vasK* (*VCA0120;* entire ORF) deleted; Rif^R^	MB_9954	This study
A1552Δ*vxrB*- Tn*vxrB* (TnVxrB)	A1552Δ*vxrB* containing mini-Tn7-*araC*-P_BAD_-*vxrB*; Rif^R^, Gent^R^	MB_9958	This study
A1552Δ*vxrC*	A1552 with *vxrC* (*VCA0567*) deleted; Rif^R^	MB_9542	This study
A1552Δ*vxrD*	A1552 with *vxrD* (*VCA0568*) deleted; Rif^R^	MB_9543	This study
A1552Δ*vxrE*	A1552 with *vxrE* (*VCA0569*) deleted; Rif^R^	MB_9544	This study
A1552Δ*oscR*	A1552 with *oscR* (*VCA0029*) deleted; TransFLP; Rif^R^	MB_3808	This study
A1552Δ*oscR* ∆*vasK*-NEW	A1552Δ*oscR* with *vasK* (*VCA0120*; entire ORF) deleted; Rif^R^	MB_9960	This study
A1552Δ*tfoX*	A1552 with *tfoX* (*VC1153*) deleted; Rif^R^	MB_45	[45]
A1552Δ*tfoX* Δ*vasK*-NEW	A1552Δ*tfoX* with *vasK* (*VCA0120*; entire ORF) deleted; Rif^R^	MB_9961	This study
A1552Δ*qstR*	A1552 with *qstR* (*VC0396*) deleted; Rif^R^	MB_600	[33]
A1552Δ*qstR* Δ*vasK*-NEW	A1552Δ*qstR* with *vasK* (*VCA0120*; entire ORF) deleted; Rif^R^	MB_9962	This study
A1552Δ*tfoY*	A1552 with *tfoY* (*VC1722*) deleted; Rif^R^	MB_828	[28]
A1552Δ*tfoY* Δ*vasK*-NEW	A1552Δ*tfoY* with *vasK* (*VCA0120*; entire ORF) deleted; Rif^R^	MB_9963	This study
A1552Δ*hapR*	A1552 with *hapR* (*VC0583*) deleted; TransFLP; Rif^R^	MB_2620	[24]
A1552Δ*hapR* Δ*vasK*-NEW	A1552Δ*hapR* with *vasK* (*VCA0120*; entire ORF) deleted; Rif^R^	MB_9964	This study
A1552Δ*vasK*-NEW	A1552 with *vasK* (*VCA0120;* entire ORF) deleted; Rif^R^	MB_9535	This study
A1552Δ*vasK*-NEW Δ*hapA*	A1552Δ*vasK*-NEW with *hapA* (*VCA0865*) deleted; TransFLP; Rif^R^	MB_9967	This study
A1552Δ*vasK*-NEW-Tn*tfoX-strep* (TnTfoX)	A1552Δ*vasK*-NEW containing mini-Tn7-*araC*-P_BAD_-*tfoX*-strep; Rif^R^, Gent^R^	MB_9968	This study
A1552Δ*vasK*-NEW-Tn*tfoY-strep* (TnTfoY)	A1552Δ*vasK*-NEW containing mini-Tn7-*araC*-P_BAD_-*tfoY*-strep; Rif^R^, Gent^R^	MB_9969	This study
A1552-T6SS [SNP-conv]	A1552 with SNP45 in intergenic region between *VCA0106* and *VCA0107* (*vipA*) converted from G to T by site-directed mutagenesis; Rif^R^	MB_9063	[36]
A1552-T6SS [SNP-conv] Δ*vasK*-NEW	A1552-T6SS[SNP**-**conv] with *vasK* (*VCA0120*; entire ORF) deleted; Rif^R^	MB_9536	This study
A1552-T6SS [SNP-conv] Δ*hapA*	A1552-T6SS[SNP**-**conv] with *hapA* (*VCA0865*) deleted; Rif^R^	MB_9308	This study
A1552-T6SS [SNP-conv]-*vipA-sfGFPv2*	A1552-T6SS[SNP**-**conv] carrying *vipA-sfGFP* translational fusion (v2: without ATG at start of *sfGFP*), TransFLP; Rif^R^	MB_9521	[36]
A1552-*vipA-sfGFP*v2	A1552 carrying *vipA-sfGFP* translational fusion (v2: without ATG at start of *sfGFP*); TransFLP; Rif^R^	MB_3909	[28]
A1552- *vipA-sfGFP*v2 Δ*vasK*-NEW	A1552-*vipA-sfGFP*v2 with *vasK* (*VCA0120*; entire ORF) deleted; Rif^R^	MB_9560	This study
A1552-*vipA-sfGFP*v2-Tn*tfoX-strep*	A1552-*vipA-sfGFP*v2 containing mini-Tn7-*araC*-P_BAD_-*tfoX-strep*; Rif^R^, Gent^R^	MB_3961	[28]
A1552-*vipA-sfGFP*v2-Tn*tfoY-strep*	A1552-*vipA-sfGFP*v2 containing mini-Tn7-*araC*-P_BAD_-*tfoY-strep*; Rif^R^, Gent^R^	MB_3962	[28]
A1552-*vipA-sfGFP*v2- chg[−10box]	A1552-*vipA-sfGFP*v2 with site-directed mutation (AA to GC) in −10 element in intergenic region between *VCA0106* and *VCA0107* (*vipA*); Rif^R^	MB_9970	This study
A1552-*vipA-sfGFP*v2 Δ*tfoX*	A1552-*vipA-sfGFP*v2 with *tfoX* (*VC1153*) deleted; Rif^R^	MB_9971	This study
A1552-*vipA-sfGFP*v2 Δ*qstR*	A1552-*vipA-sfGFP*v2 with *qstR* (*VC0396*) deleted; Rif^R^	MB_9972	This study
A1552-*vipA-sfGFP*v2 Δ*tfoY*	A1552-*vipA-sfGFP*v2 with *tfoY* (*VC1722*) deleted; Rif^R^	MB_4221	This study
A1552-*vipA-sfGFP*v2 Δ*hapR*	A1552-*vipA-sfGFP*v2 with *hapR* (*VC0583*) deleted; Rif^R^	MB_4217	[28]
A1552-*vipA-sfGFP*v2 Δ*vxrA*	A1552-*vipA-sfGFP*v2 with *vxrA* (*VCA0565*) deleted; TransFLP; Rif^R^	MB_9975	This study
A1552-*vipA-sfGFP*v2 Δ*vxrB*	A1552-*vipA-sfGFP*v2 with *vxrB* (*VCA0566*) deleted; TransFLP; Rif^R^	MB_9976	This study
A1552-*vipA-sfGFP*v2 Δ*vxrB*-TnVxrB	A1552-*vipA-sfGFP*v2Δ*vxrB* containing mini-Tn7-*araC*-P_BAD_-*vxrB*; Rif^R^, Gent^R^	MB_9977	This study
A1552-*vipA-sfGFP*v2 Δ*vxrC*	A1552-*vipA-sfGFP*v2 with *vxrC* (*VCA0567*) deleted; Rif^R^	MB_9549	This study
A1552-*vipA-sfGFP*v2 Δ*vxrD*	A1552-*vipA-sfGFP*v2 with *vxrD* (*VCA0568*) deleted; Rif^R^	MB_9959	This study
A1552-*vipA-sfGFP*v2 Δ*vxrE*	A1552-*vipA-sfGFP*v2 with *vxrE* (*VCA0569*) deleted; Rif^R^	MB_9551	This study
A1552-*vipA-sfGFP*v2 Δ*oscR*	A1552-*vipA-sfGFP*v2 with *oscR* (*VCA0029*) deleted; TransFLP; Rif^R^	MB_9978	This study
A1552-*vipAsfGFP*v2 ∆*tsrA*	A1552-*vipA-sfGFP*v2 with *tsrA* (*VC0070*) deleted; TransFLP; Rif^R^	MB_10110	This study
A1552-*vipAsfGFP*v2 ∆*tsrA*∆*vxrB*	A1552-*vipA-sfGFP*v2 with *tsrA* (*VC0070*) and *vxrB* (*VCA0566*) deleted; TransFLP; Rif^R^	MB_10112	This study
N16961	Wild-type; O1 El Tor Inaba isolated in 1975 in Bangladesh; *hapR* with frameshift mutation; Strep^R^	MB_2	[74]
N16961Δ*vasK*-NEW	N16961 with *vasK* (*VCA0120;* entire ORF) deleted; Strep^R^	MB_9755	This study
N16961-*vipA-sfGFP*v2	N16961 carrying *vipA-sfGFP* translational fusion (v2: without ATG at start of *sfGFP*); Strep^R^	MB_9979	This study
C6706 (Strep^S^) (original)	Wild-type; O1 El Tor Inaba collected in 1991 in Peru; original isolate before introduction of streptomycin resistance mutation; non-mutated *luxO;* Strep^S^	MB_4522	[64]
C6706 (Strep^S^) Δ*vasK*-NEW	C6706 (Strep^S^) with *vasK* (*VCA0120*; entire ORF) deleted; Strep^S^	MB_9757	This study
C6706 (Strep^S^)-*vipA-sfGFP*v2	C6706 (Strep^S^) carrying *vipA-sfGFP* translational fusion (v2: without ATG at start of *sfGFP*); Strep^S^	MB_9980	This study
C6706 (Strep^R^)	C6706 with *luxO* mutation (G997A resulting in G333S); Strep^R^	MB_4524	[64]
C6706 (Strep^R^) Δ*vasK*-NEW	C6706 (Strep^R^) with *vasK* (*VCA0120;* entire ORF) deleted; Strep^R^	MB_9758	This study
C6706 (Strep^R^)-*vipA-sfGFP*v2	C6706 (Strep^R^) carrying *vipA-sfGFP* translational fusion (v2: without ATG at start of *sfGFP*); Strep^R^	MB_9981	This study
C6709	Wild-type; O1 El Tor Inaba collected in 1991 in Peru; Strep^R^	MB_1503	[75]
C6709Δ*vasK*-NEW	C6709 with *vasK* (*VCA0120;* entire ORF) deleted; Strep^R^	MB_9759	This study
C6709-*vipA-sfGFP*v2	C6709 carrying *vipA-sfGFP* translational fusion (v2: without ATG at start of *sfGFP*); Strep^R^	MB_9982	This study
E7946	Wild-type; O1 El Tor Ogawa isolated in 1978 in Bahrain; Strep^R^	MB_2600	[76]
E7946Δ*vasK*-NEW	E7946 with *vasK* (*VCA0120;* entire ORF) deleted; Strep^R^	MB_9760	This study
E7946-*vipA-sfGFP*v2	E7946 carrying *vipA-sfGFP* translational fusion (v2: without ATG at start of *sfGFP*); Strep^R^	MB_9983	This study
P27459	Wild-type; O1 El Tor Inaba isolated in 1976 in Bangladesh; Strep^R^	MB_1504	[77]
P27459Δ*vasK*-NEW	P27459 with *vasK* (*VCA0120*; entire ORF) deleted; Strep^R^	MB_9761	This study
P27459-*vipA-sfGFP*v2	P27459 carrying *vipA-sfGFP* translational fusion (v2: without ATG at start of *sfGFP*); Strep^R^	MB_9984	This study
*** E. coli ***			
MC4100-TnKan	MC4100 containing mini-Tn7-*aph* (TnKan); Strep^R^, Kan^R^, Gent^R^	MB_4118	This study
TOP10-TnKan	TOP10 containing mini-Tn7-*aph* (TnKan); Strep^R^, Kan^R^, Gent^R^	MB_4119	[28]
MG1655-Strep	MG1655 exposed to UV light (10 s) and selected on Strep-containing plates; Strep^R^	MB_4453	This study
S17-1λpir	Tp^R^ Sm^R^ *recA thi pro* hsdR2M1 RP4 : 2-Tc:Mu:Km^R^ Tn7 (λpir); Strep^R^	MB_648	[78]
**Plasmids**			
pBR-FRT-Kan-FRT2	pBR322 derivative containing improved FRT-*aph*‐FRT cassette; used as template for TransFLP; Amp^R^, Kan^R^	MB_3782	[28]
pBR-flp	pBR322 derivative containing FLP+, λ cI857+, λ *p*R from pCP20 integrated into the *Eco*RV site of pBR322; Amp^R^	MB_1203	[46]
pGP704-Sac28	Suicide plasmid, *oriR6K sacB;* Amp^R^	MB_649	[49]
pGP704-mTn*tfoX-strep*	pGP704 with mini-Tn7 carrying *araC* and P_BAD_-driven *tfoX-strep;* Amp^R^, Gent^R^	MB_3664	[28]
pGP704-mTn*tfoY-strep*	pGP704 with mini-Tn7 carrying *araC* and P_BAD_-driven *tfoY-strep;* Amp^R^, Gent^R^	MB_2941	[28]
pGP704-mTnVxrB	pGP704 with mini-Tn7 carrying *araC* and P_BAD_-driven *vxrB;* Amp^R^, Gent^R^	MB_9955	This study
p28-*tfoX* (pGP704-Sac28 Δ*tfoX*)	pGP704-Sac28 carrying a gene fragment resulting in a deletion within *tfoX* (*VC1153*); Amp^R^	MB_1013	[45]
pGP704-28-SacB∆*qstR*	pGP704-Sac28 carrying a gene fragment resulting in a deletion within *qstR* (*VC0396*); Amp^R^	MB_1118	[33]
pGP704-28-SacBΔ*tfoY*	pGP704-Sac28 carrying a gene fragment resulting in a deletion within *tfoY* (*VC1722*); Amp^R^	MB_4116	[28]
p28-*hapR* (pGP704-Sac28 Δ*hapR*)	pGP704-Sac28 carrying a gene fragment resulting in a deletion within *hapR* (*VC0583*); Amp^R^	MB_1038	[45]
pGP704-Sac28 Δ*vasK*-NEW	pGP704-Sac28 carrying a gene fragment resulting in deletion of *vasK* (*VCA0120*; entire ORF); Amp^R^	MB_9533	This study
pGP704-Sac28∆*vxrC*	pGP704-Sac28 carrying a gene fragment resulting in a deletion within *vxrC* (*VCA0567*); Amp^R^	MB_9556	This study
pGP704-Sac28∆*vxrD*	pGP704-Sac28 carrying a gene fragment resulting in a deletion within *vxrD* (*VCA0568*); Amp^R^	MB_9557	This study
pGP704-Sac28∆*vxrE*	pGP704-Sac28 carrying a gene fragment resulting in a deletion within *vxrE* (*VCA0569*); Amp^R^	MB_9558	This study
pGP704-Sac28-*vipA-sfGFP*v2::FRT	pGP704-Sac28 carrying a gene fragment resulting in a *vipA-sfGFP* translational fusion (v2: without ATG at start of *sfGFP*); Amp^R^	MB_9957	This study
pGP704-Sac-Kan	Suicide plasmid, *oriR6K sacB*; Kan^R^	MB_6038	[29]
pGP704-Sac-Kan-chg[−10box] [SNP45-G]	pGP704-Sac-Kan carrying a genome fragment resulting in a site-directed mutation in the −10 element (AA to GC) located in the intergenic region between *VCA0106* and *VCA0107* (*vipA*) with a SNP45 as ‘G’; Kan^R^	MB_9664	[36]

*Reference locus tags of reference strain N16961 according to the literature [74].

### Recombinant DNA techniques and genetic engineering

DNA manipulations and molecular cloning were performed using standard methods. PCR amplifications were performed using GoTaq (Promega), Pwo (Roche) or Expand High Fidelity (Roche) polymerases according to the suppliers’ recommendations. Genetic engineering of *V. cholerae* was performed using either natural transformation and FLP recombination (TransFLP) [46–48] or allelic exchange using the counterselectable plasmids pGP704-Sac28 or pGP704-Sac-Kan delivered via biparental mating from *E. coli *, as previously described [28, 49]. All constructs were checked by colony PCR and Sanger sequencing (Microsynth). Sequences were analysed using SnapGene version 4.3.11 and Geneious Prime. A mini-Tn7 transposon carrying *araC* and the gene of interest under control of the *P*_BAD_ promoter was integrated into a neutral chromosomal locus downstream of *glmS* in *V. cholerae* by triparental mating, as previously described [50].

### Interbacterial killing assay

Bacterial killing was assessed following a previously established assay [24]. The conditions used in the experiment were optimized to observe sporadic T6SS activity, and the final method is described below. The respective predators and *V. cholerae* prey (A1552Δ4EI-Kan) were grown in glass tubes at 180 r.p.m. and 30 °C for 3 h, concentrated to an OD_600_ value of 10, mixed at a 1 : 1 ratio and spotted onto filters on prewarmed LB agar plates (containing 0.2 or 0.02 % arabinose, where indicated in the figure legends). After 4 h of incubation at 30 °C, the bacteria were resuspended, serially diluted and plated onto kanamycin-containing LB agar plates to enumerate colony-forming units (shown as log-transformed c.f.u. ml^−1^ in the graphs). Experiments were performed at least three times independently. Statistical significance was determined using GraphPad Prism 9.1.1 (for MacOS) for log-transformed data [51] using a one-way or two-way ANOVA followed by Šídák’s, Tukey’s or Dunnett’s multiple comparisons tests, as indicated in the figure legends. If no prey bacteria were recovered, the detection limit was used to calculate the mean of the set of independent experiments and to perform statistical analysis.

### Imaging of T6SS sheath structures

To image T6SS sheath structures in strains carrying the translational fusion *vipA-sfGFP*, cells were immobilized on microscope slides coated with an agarose pad (1.2 % in 0.5× PBS), covered with a coverslip, and observed in phase contrast and epifluorescence mode (green channel) using a Zeiss LSM 700 inverted confocal laser scanning microscope with an attached HXP 120 light unit (Zeiss). Images were adjusted for contrast and brightness and cropped using Fiji software [52]. The images are representative of at least three biologically independent replicates.

Movies were taken by imaging the cells every minute for 30 min. The drift of the movie was corrected using the HyperStackReg plugin (version 5.6, doi:10.5281/zenodo.2252521) of Fiji. Brightness and contrast were adjusted to make the T6SS structures clearly visible. All images of the GFP channel were stacked using the Z-project function of Fiji (Projection type: ‘max intensity’). This overlay was merged with the last picture of the phase contrast channel to obtain the image shown in Fig. 2(c) below.

### DenoiSeg model for segmentation of bacteria in phase contrast images

For the segmentation of bacteria, a ‘few-shot learning’ DenoiSeg [53] model was trained on the phase contrast channel of the images of bacteria (available on Zenodo; doi: 10.5281/zenodo.7467554). Ground truth annotations were manually drawn in QuPath v0.3 [54] for 11 images representing low and high bacterial cell densities, with varying shapes and different contrast conditions, which was applied to encompass the variability of the raw data. Annotated images were normalized and background-corrected by dividing each image by a 200-pixel Gaussian-blurred version. Corrected images were split into 44 patches (256×256 pixels each) of which 33 patches served as the training set and 11 patches as a validation set. Both sets were augmented with rotated versions of the images. The model was trained for 20 epochs using the default settings of DenoiSeg, whereby an average validation loss of 0.2 was already reached at 15 epochs. Raw data and ground truth annotations are accessible in a separate repository (Zenodo; doi: 10.5281/zenodo.7467196).

### Image analysis using ImageJ/Fiji script

The total number of bacteria in each image was counted automatically. The input for this analysis was the binary file that was generated using the DenoiSeg model described above with the help of a custom-made Fiji script (Zenodo; doi: 10.5281/zenodo.7467554). Briefly, the DenoiSeg model was run, and the probability images for the foreground (bacteria) and the one-hot (bacterial edges) class were retrieved as the model’s output. The one-hot probability was subsequently subtracted from the foreground probability image, and the resulting image was thresholded to generate a bacterial mask image. The resulting mask images were saved as individual tiff files for downstream batch analysis. This analysis was performed using the MicrobeJ plugin [55] of the Fiji software with a minimum size threshold of 0.5 µm^2^. The number of bacteria containing GFP-tagged T6SS sheath structure(s) was counted manually after equal adjustment of the brightness and contrast of the GFP channel in each image using Fiji software.

### Western blotting

To investigate the production of the T6SS inner tube protein Hcp, cell lysates were prepared as described previously [28]. In brief, overnight cultures were diluted 1 : 100 in LB medium and grown in glass tubes at 180 r.p.m. and 30 °C for 3 or 7.5 h. Cells were harvested by centrifugation, and the bacterial pellet was resuspended in 2× Laemmli buffer (Sigma-Aldrich), adjusting for the total number of bacteria according to the cultures’ optical density at 600 nm (OD_600_). To check for T6SS-secreted Hcp, 1.5 or 12.5 ml of the culture supernatant was filter-sterilized (0.2 µm filter; VWR), and the proteins (from 900 µl or 9 ml of filtered supernatant) were precipitated using trichloroacetic acid (TCA). The precipitated proteins were washed with acetone before resuspension in 30 µl of 2× Laemmli buffer (Sigma-Aldrich). All samples were heated at 95 °C for 15 min.

Proteins were separated on 12 % Mini-PROTEAN TGX Stain-Free precast gels and transferred onto PVDF membranes using a Trans-Blot Turbo Transfer System (Bio-Rad) according to the manufacturer’s instructions. Primary antibodies against Hcp (raised against synthetic peptides; Eurogentec #1510528 [28]) were diluted 1 : 5 000, while the secondary anti-rabbit IgG horseradish peroxidase (HRP) antibody (Sigma-Aldrich, A9169) was diluted 1 : 20 000. Loading controls were performed using the anti-Sigma70-HRP conjugate (BioLegend, 663205) at a 1 : 10 000 dilution. Lumi-Light^PLUS^ Western Blotting Substrate (Roche) served as the HRP substrate. The signal was detected using a ChemiDoc XRS+station (Bio-Rad).

## Results and discussion

### Defining *in vitro* conditions that allow the detection of T6SS-mediated killing by 7PET strain A1552

**Fig. 1.**
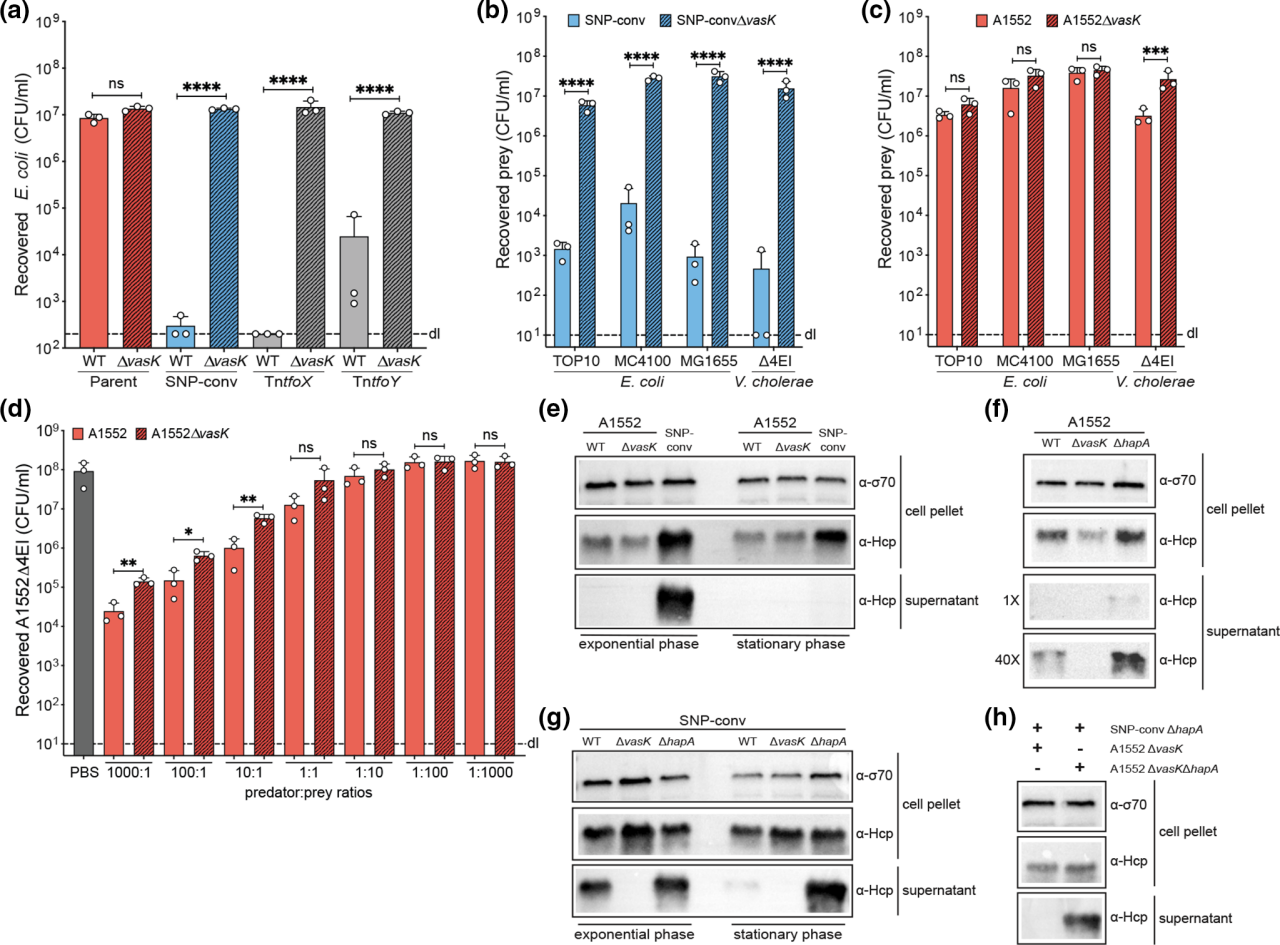
Experimental conditions impact the detectability of T6SS activity. (**a–d**) Interbacterial killing assays using different *E. coli* strains or *V. cholerae* strain A1552∆4EI as prey. Predator and prey were mixed at a 10 : 1 ratio (**a–c**) or at a range of ratios (**d**). Bacterial cultures were grown for 3 h at 30 °C before being mixed and spotted onto a solid surface, and these plates were incubated at 37 °C for 4 h (a–d; +0.2 % arabinose for panel a). Numbers of surviving prey are depicted on the *y*-axis (c.f.u. ml^−1^) and the bars represent the average of three independent biological replicates (±sd), as indicated by individual dots. dl, Detection limit. Statistical significance is shown using one-way (**a, d**) or two-way (**b, c**) ANOVA followed by Tukey’s (**a**) or Šídák’s (**b–d**) multiple comparisons tests. Strains were compared to their ∆*vasK* counterparts. *, *P*<0.05; **, *P*<0.01; ***, *P*<0.001; ****, *P*<0.0001; n.s., not significant. (**e–h**) T6SS activity was scored by Western blot detection of the secreted T6SS tube protein Hcp. (**e**) Hcp production and secretion during growth to exponential and stationary phases in wild-type A1552 (WT) or the ∆*vasK* and SNP-converted variants. (**f**) Hcp production and secretion in WT, ∆*vasK,* and ∆*hapA* strains. For the 40× conditions, 10× volumes of supernatant were used for precipitation and 4× sample volumes were loaded onto the gel. (**g, h**) Absence of Hcp in the supernatant of a stationary-phase SNP-converted strain is HA/protease-dependent. Hcp production and secretion in an SNP-converted strain and its ∆*vasK* or ∆*hapA* mutants (**g**) and in co-cultures of an SNP-converted ∆*hapA* strain with A1552∆*vasK* (left) or A1552∆*vasK*∆*hapA* (right) (**h**).

While TfoX- and TfoY-dependent T6SS regulation has been extensively studied in 7PET strains, in-depth information on the T6SS behaviour of these strains under standard laboratory conditions is still lacking. We therefore compared T6SS-mediated interbacterial killing activity in the WT representative 7PET strain A1552 and several of its derivatives, which served as controls. Precisely, these derivatives included an SNP-converted variant of strain A1552 that is known to constitutively produce T6SS structures *in vitro* as mentioned above [36] as well as TfoX-/TfoY-expressing variants, which have been characterized for their T6SS activity in previous work [24, 28]. Mutants of each strain that lacked the T6SS membrane complex protein VasK (∆*vasK*) served as negative controls. As shown in Fig. 1(a), no statistically significant killing was observed in an interbacterial competition assay for the parental WT and T6SS-deficient strains, while the prey population was between ~500 and 75 000-fold depleted by the other derivates (Fig. 1a). This result was surprising to us, as different groups had observed T6SS-mediated killing *in vitro* for 7PET strains [38, 39, 56], as described in the Introduction. Since different groups use different interbacterial killing conditions, we tried to vary our experimental conditions to see if this would allow us to detect T6SS killing by *V. cholerae* 7PET strain A1552.

As different research groups tend to use different prey strains, which is often dictated by the antibiotic resistance profiles of the predator and prey, we repeated the experiment using either the *E. coli* strain TOP10 as prey as above or, alternatively, two other prominent *E. coli* strains, MC4100 or MG1655. In addition, we also tested a T6SS-sensitive *V. cholerae* as prey. T6SS sensitivity was obtained in this strain by deleting its four effector-immunity gene clusters (∆4EI), and selection was achieved through chromosomal insertion of a kanamycin resistance cassette. These three prey strains were killed by the SNP-converted A1552 strain, which served as a positive control, at levels similar to those of *E. coli* TOP10 (Fig. 1b). For WT A1552, a slight killing tendency was observed toward some *E. coli* prey, but this was not statistically significant (Fig. 1c). In contrast, compared to that for its T6SS-deficient mutant, a statistically significant reduction in survival was observed for the ∆4EI *V. cholerae* prey when it was competed with the WT strain. We concluded that the 7PET strain was able to kill other bacteria but that this T6SS-mediated killing activity led solely to a minor depletion of the prey, which was often at the limit of statistical significance (as shown here for *E. coli* as prey).

Next, we tested different predator-to-prey ratios, another important variable between experiments from different research groups, to see if this could influence the outcome of these killing assays. Notably, the measured values were again highly variable between the biologically independent experiments (Fig. 1d) and also between different sets of experiments in which the WT served as a control (see other data below). Hence, based on these results, we concluded solely that an excess of prey probably masked residual T6SS killing ability and therefore went on to perform the experiments described below with an equal stoichiometry between predator and prey.

### Low level of T6SS-secreted Hcp protein is detectable in *V. cholerae* A1552

Given the low but significant killing ability under laboratory conditions, we next tested the 7PET strain’s T6SS activity using a secondary readout taking the mode of action of T6SSs into consideration. Briefly, these nanomachines belong to the group of contractile injection systems [57]. As such, they consist of a double tubular structure, whereby contraction of an outer sheath tube triggers the ejection of an inner tube and its toxin-decorated tip [11]. The inner tube is composed of Hcp protein hexamer stacks, rendering Hcp a marker protein for secretion. Hence, we aimed to detect intracellular and secreted Hcp after we grew the *V. cholerae* WT and *vasK*-deficient mutant to the exponential or stationary phase. As shown in Fig. 1(e), we were unable to detect the protein in the supernatant of cultures of both strains, consistent with previous work [36, 44]. Hcp production inside the cells was observed, although at reduced levels compared to those in the SNP-converted positive control strain, which also efficiently secreted the inner tube protein into the supernatant (Fig. 1e).

Given that we determined that the killing efficiency of the WT 7PET strain was 3–5 log lower than that of the SNP-converted positive control strain (Fig. 1b, c), we scaled up the secretion assay by a factor of 40, which ultimately allowed us to also detect secreted Hcp for the WT strain (Fig. 1f). Collectively, these secretion data support the idea that the 7PET strains show low levels of T6SS activity under laboratory conditions.

### HA/protease masks T6SS activity in quorum-sensing proficient *V. cholerae* strains

While testing Hcp secretion, we noted an unexpected phenotype for the SNP-converted positive control strain. In contrast to that in the exponential phase conditions, the Hcp protein was undetectable in the culture supernatant when the bacteria were grown to stationary phase (Fig. 1e). This observation was surprising, as intracellular Hcp levels were maintained at levels almost equal to those during the exponential phase, consistent with previous studies that reported high intracellular Hcp levels at an OD_600_ of >2.0 [38, 56]. Indeed, Ishikawa and colleagues suggested that the presence of Hcp late during growth was due to positive regulation of the *hcp* genes by the master regulator of QS, HapR, at high cell density [56]. In addition, a lack of secretion and therefore putative intracellular accumulation of Hcp would be expected to downregulate Hcp production [58]. Given that HapR regulates the expression of the HA/protease-encoding gene *hapA* [59]*,* we wondered whether the extracellular protease interfered with Hcp detection. We therefore repeated the Hcp secretion assay in the SNP-converted genetic backgrounds of the WT, ∆*vasK* and ∆*hapA* strains. As shown in Fig. 1(g), secreted Hcp remained detectable in cultures of the *hapA*-deficient strain during growth to stationary phase.

To further support the idea that the HA/protease degrades extracellular Hcp instead of acting on the T6SS intracellularly or in the periplasm, which is unlikely given its type II secretion system dependency [60], we co-incubated the SNP-converted Hcp-secreting ∆*hapA* strain with either HapA-producing or HapA-nonproducing T6SS-deficient bacteria. This experiment showed that extracellular complementation occurred, which resulted in extracellular Hcp degradation by HA/protease (Fig. 1h). Consistent with this finding, secreted Hcp was better detectable in the parental strain A1552 when it was *hapA*-deficient, especially under the scale-up conditions mentioned above (Fig. 1f). Our data therefore demonstrate that Hcp secretion assays might not properly reflect the T6SS’s activity status when performed with QS-proficient and therefore HA/protease-producing *V. cholerae* strains.

### Sporadic T6SS production occurs in 7PET strain A1552

**Fig. 2.**
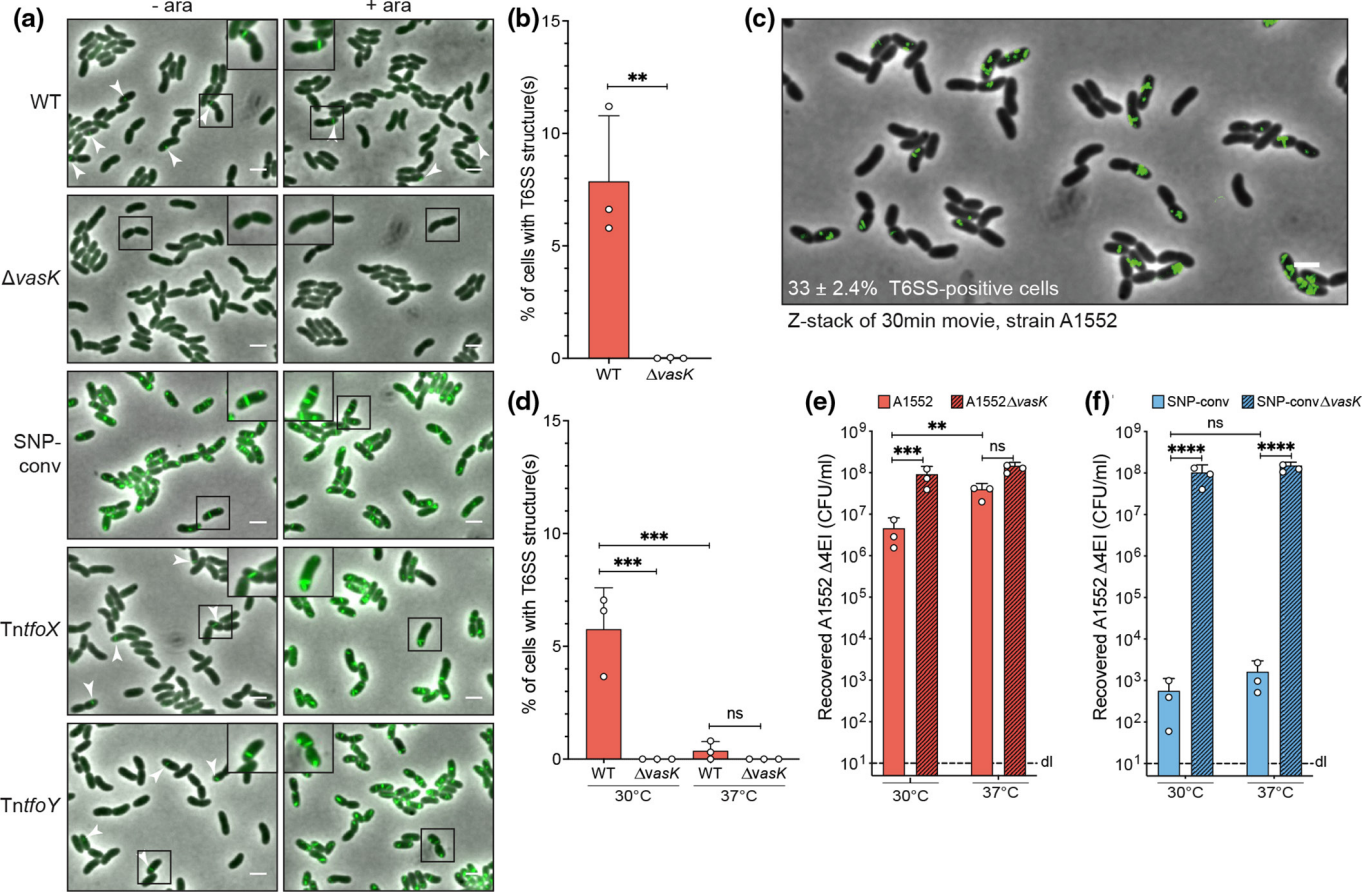
Sporadic T6SS production in 7PET strain A1552. (**a**) Visualization of T6SS structures in strains carrying a translational fusion of the T6SS sheath protein VipA and sfGFP. Wild-type 7PET strain A1552 is compared to a T6SS mutant (∆*vasK*), the SNP-converted variant, or strains that carry arabinose (ara)-inducible TfoX and TfoY constructs on a transposon (Tn*tfoX* and Tn*tfoY*). Bacteria were imaged after growth in the absence (-ara) or presence (+ara) of 0.2 % ara. Experiments were repeated three times. Micrographs are a merge of the green (GFP) and phase contrast channels. White arrowheads indicate unexpected sporadic VipA-sfGFP foci/sheath structures. Bars, 2 µm. A zoomed image of the boxed region is shown in the corner of each micrograph with increased contrast settings to improve visualization of the localized GFP signal. (**b, d**) Quantification of T6SS structures in images of A1552 WT and ∆*vasK* grown at 30 °C (**b**) or at 30 and 37 °C (**d**). The proportion of cells with at least one T6SS structure is indicated. Bars represent the average of three independent biological replicates (±sd), as indicated by individual dots. (**c**) Strain A1552 carrying the VipA-sfGFP translational fusion was imaged every minute for 30 min. An overlay of the 31 images of the GFP channel was merged onto the last phase contrast image. The percentage of cells with at least one T6SS structure during the time-lapse was counted on the merged image of three independent replicates and the average±sd is shown in the lower left corner of the image. Bar, 2 µm. (**e, f**) Temperature impacts sporadic T6SS activation. The impact of temperature on T6SS killing was tested in an interbacterial killing assay by pre-growing and co-incubating predator and prey cells at a ratio of 1 : 1 on plates at either 30 or 37 °C, as depicted on the *x*-axis. The wild-type (**e**) or SNP-converted strain (**f**) and their *vasK*-deficient mutants were analysed. Numbers of surviving prey are depicted on the *y*-axis (c.f.u. ml^-1^) and each bar represents the average of three independent biological replicates (±sd), as indicated by individual dots. dl, Detection limit. Statistical significance is shown using an unpaired *t*-test (**b**), a one-way (**d**) or two-way (**e, f**) ANOVA followed by Šídák’s (**d**) or Tukey’s (**e, f**) multiple comparisons tests. **, *P*<0.01; ***, *P*<0.001; ****, *P*<0.0001; n.s., not significant.

To better understand the nature of the low T6SS activity in 7PET strains, we directly imaged the machinery using a translational fusion of the sheath protein VipA and superfolder GFP (sfGFP) [23, 24, 28]. Initially, this approach seemed to support T6SS silencing, especially when compared to the SNP-converted [36] or TfoX-/TfoY-expressing derivates as positive controls (Fig. 2a). However, upon closer inspection of the images, we observed few bacteria within the bacterial population with green fluorescent foci (probably presenting contracted sheath structures) and, even more rarely, cells with elongated T6SS sheaths, a phenotype that was not observed in the ∆*vasK* mutant control (Fig. 2a). This latter finding is in line with the essentiality of the membrane complex component VasK (also known as TssM in other organisms [61]) for T6SS sheath assembly and, accordingly, the absence of assembled T6SS structures in a *vasK* mutant of the constitutive T6SS-active strain 2740-80 [62].

We quantified those T6SS-positive cells in snapshot images or after time-lapse microscopy, which showed that only a small fraction (~5–10 %; Fig. 2b) of the population had T6SS structure(s), while over time, up to one-third of cells sporadically produced the machinery (Fig. 2c and Movie S1, available with the online version of this article). At this point, we cannot exclude that the residual cells within the population also produce T6SS structure(s) eventually, yet we consider it unlikely that such T6SS production would occur simultaneously in the entire population. Notably and consistent with the aim of this study, our data demonstrate that the T6SS is indeed active in 7PET strains, although only in a small subfraction of the population concomitantly. Known T6SS-inducing conditions, on the other hand, such as the production of TfoX/TfoY or SNP conversion, led to uniform T6SS production, consistent with what has been reported previously (Fig. 2a) [24, 28, 36].

### Temperature impacts sporadic T6SS production

Next, we evaluated the impact of temperature on sporadic T6SS activation. This parameter is especially important when comparing the environmental lifestyle of *V. cholerae* strains in endemic areas such as the Bay of Bengal (with sea surface temperatures reaching up to 30 °C [63]) with the pathogen’s infectious cycle in humans (with a temperature of 37 °C). Interestingly, we observed significantly fewer T6SS structures in those bacteria that were grown at 37 °C than in those grown at 30 °C (Fig. 2d). Consistently, we recovered more prey in the interbacterial killing assay when the predator and prey were pre-grown in broth and competed on plates at 37 ˚C compared to the 30 ˚C setup (Fig. 2e). The T6SS killing activity of the SNP-converted positive control strain, on the other hand, appeared to be temperature-insensitive (Fig. 2f), suggesting that the sporadic T6SS production of the 7PET strains was temperature-dependent but not the T6SS assembly or secretion processes.

### Sporadic T6SS production occurs in several 7PET strains and relies on a functional QS circuit

**Fig. 3.**
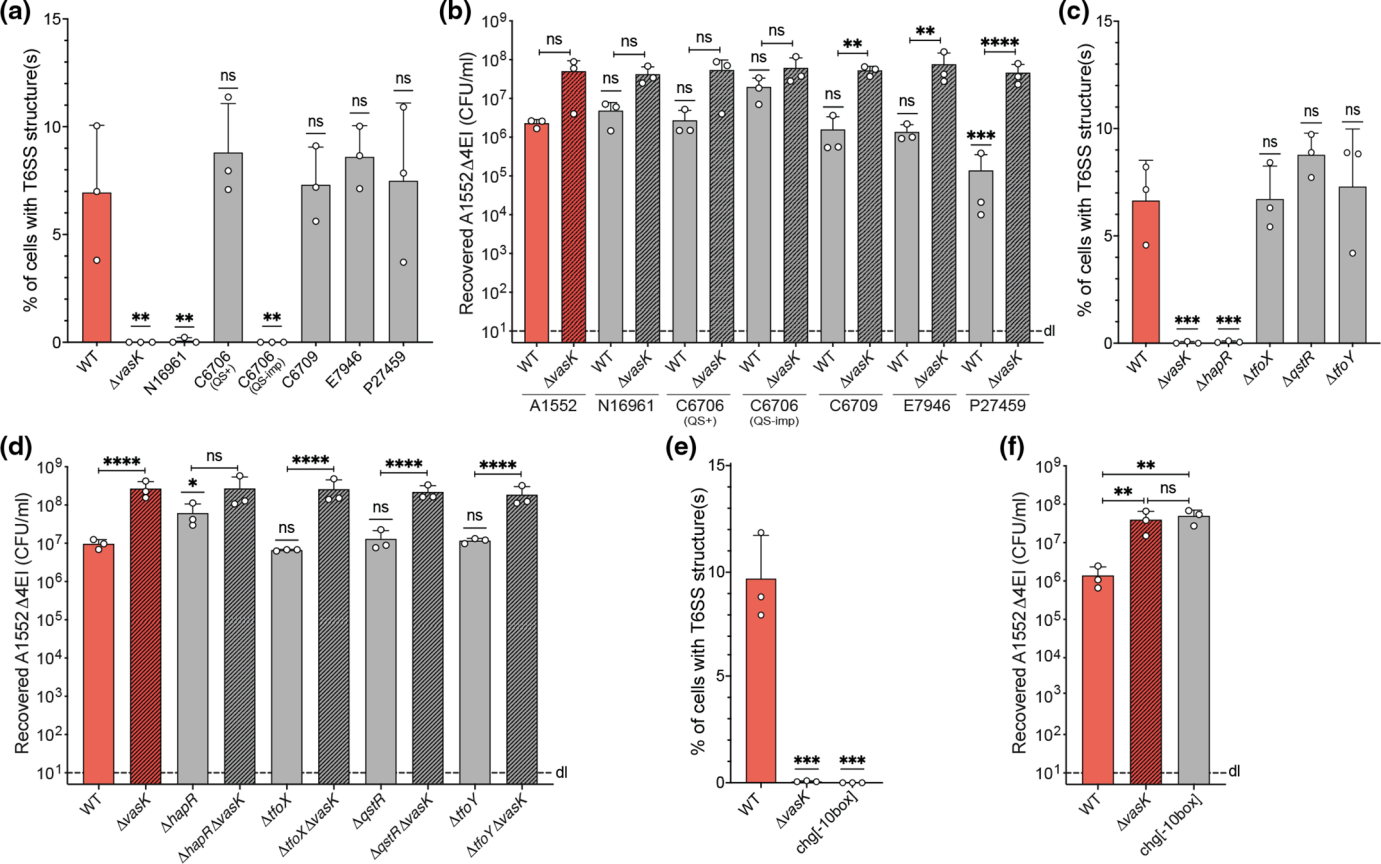
Sporadic T6SS production occurs in most 7PET strains and depends on an intact QS circuit. Experiments were performed with the following strains: (**a, b**) *V. cholerae* strains isolated from the seventh cholera pandemic – strains N16961 and C6706 (QS-imp) have defective and impaired QS, respectively; (**c, d**) variants of the 7PET representative strain A1552 lacking the genes encoding known T6SS regulators HapR, TfoX, QstR and TfoY; (**e, f**) strains lacking the putative promoter upstream of the T6SS main cluster. (**a, c, e**) Counting of T6SS structures in diverse strains carrying a VipA-sfGFP translational fusion. Graphs show the proportion of cells with at least one T6SS structure. Each bar depicts the average of three independent biological replicates (±sd), as indicated by individual dots. (**b, d, f**) Interbacterial killing assays using the indicated predator strains with predator–prey ratios of 1 : 1 and the growth temperature of 30 °C. Numbers of surviving prey are depicted on the *y*-axis (c.f.u. ml^−1^) and the bars represent the average of three independent biological replicates (±sd), as indicated by individual dots. d.l., Detection limit. Statistical significance using one-way ANOVA followed by Dunnett’s (**a, c, e**) or Šídák’s (**b, d, f**) multiple comparisons tests is indicated. If not otherwise indicated by brackets, the strains were always compared to the WT A1552 strain. *, *P*<0.05; **, *P*<0.01; ***, *P*<0.001; ****, *P*<0.0001; n.s., not significant.

As the data described above were based on the same strain that was used in previous studies (e.g. A1552 [38, 39, 56]), we wondered whether the phenotype was conserved in other 7PET isolates. We therefore repeated our assays with a panel of different 7PET strains that were isolated over several decades from cholera patients worldwide (see Table 1 for detailed strain descriptions). Interestingly, strains with known QS deficiencies (e.g. N16961 and a QS-impaired C6706 isolate [64]) showed no sporadic T6SS structures, while QS-proficient strains mirrored the results described for strain A1552 (Fig. 3a). Despite not always being statistically significant, which emphasizes the low level of T6SS activity, the overall killing pattern mostly recapitulated this observation. Indeed, the QS-proficient strains [C6706 (QS+), C6709, E7946 and P27459] were able to reduce the prey numbers to similar levels as strain A1552 (Fig. 3b). In contrast, killing with the C6706 QS-impaired strain was abrogated, consistent with the microscopy data (Fig. 3a). However, despite the inability to detect T6SS sheath structures (Fig. 3a), prey killing by the QS-negative strain N16961 was similar to what was observed for strain A1552 (Fig. 3b). The difference between the two readouts (i.e. the T6SS structure imaging versus the interbacterial killing assay) might be the result of the different growth conditions and differences in the two assays’ durations, as a single timepoint was assayed for imaging under liquid growth conditions, while the interbacterial killing assay reports the prey recovery after several hours of co-incubation with the predator on solid agar plates, which might partially overcome the strains’ QS deficiency.

To more directly probe the requirement of a functional QS circuit, we next tested a *hapR* mutant of strain A1552. As shown in Fig. 3c, this mutant no longer produced sporadic T6SS structures and was unable to significantly deplete the prey population (Fig. 3d). We therefore concluded that HapR activity enhances the sporadic T6SS production in 7PET strain A1552, probably due to its direct impact on auxiliary gene clusters 1 and 2 [44, 56, 65].

Given this requirement of HapR for T6SS production, we wondered whether the observed phenotype might reflect sporadic activation of the TfoX-dependent pathway even in the absence of its usual inducer chitin. Hence, we tested a *tfoX* and a *qstR* mutant, which behaved, however, indistinguishably from the WT strain, refuting this idea (Fig. 3c, d). Likewise, *tfoY* deficiency had no impact on sporadic T6SS activation (Fig. 3c, d). The sporadic T6SS activation of the WT strain was, however, fully dependent on the recently identified putative promoter upstream of *vipA* [36], as a site-directed mutant no longer produced T6SS structures (Fig. 3e) and could not kill prey (Fig. 3f).

### The VxrAB TCS triggers sporadic T6SS activation

**Fig. 4.**
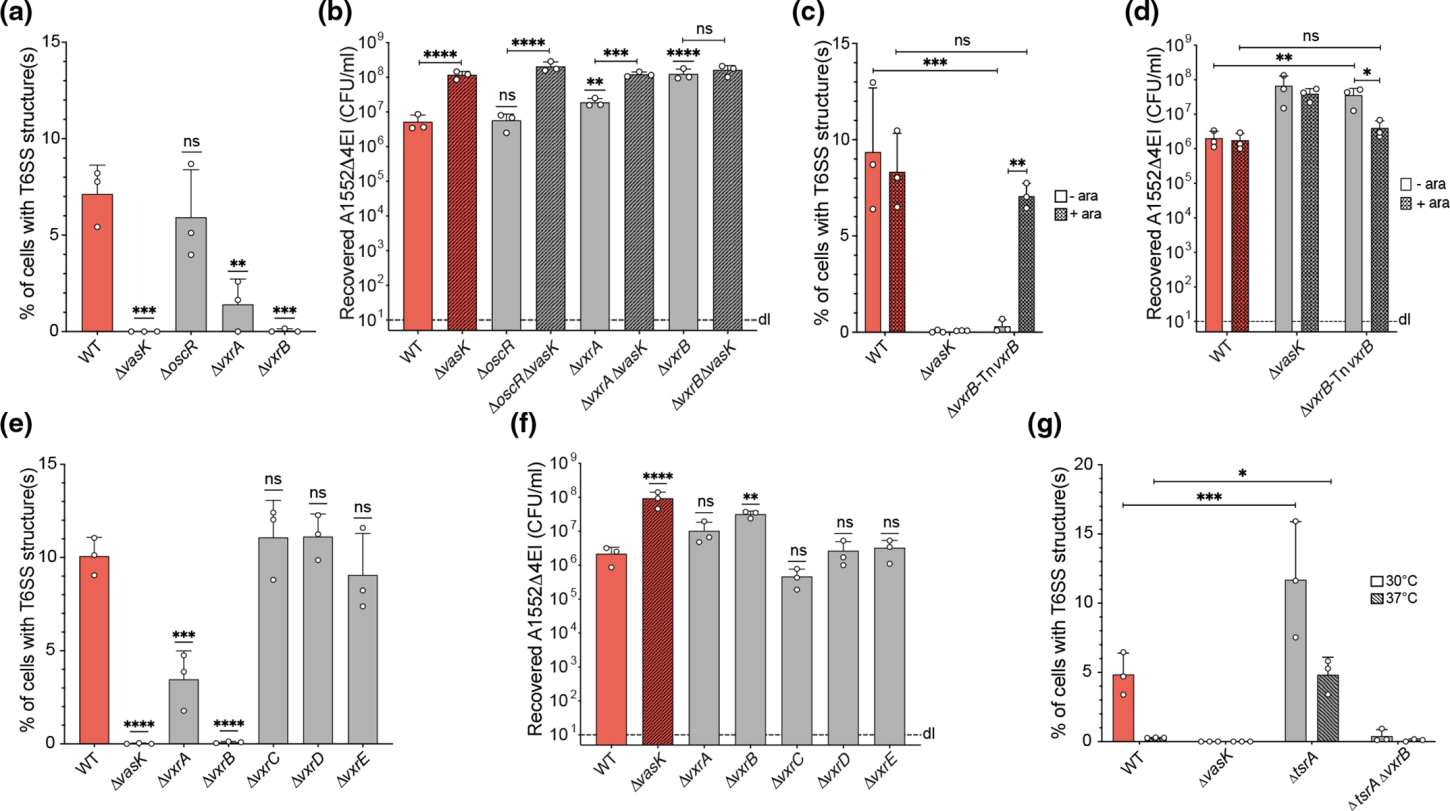
The Vxr TCS triggers T6SS activation. Counting of T6SS structures in VipA-sfGFP-carrying strains (**a, c, e, g**) or scoring interbacterial killing activity (**b, d, f**). Strains lacked *oscR,* genes of the *vxr* operon or *tsrA*, as indicated. (**c, d**) Data showing *vxrB* complementation (Tn*vxrB* depicts an ara-inducible copy of *vxrB* on a miniTn7 transposon) after growth of the strains in the absence (-ara) or presence (+0.02 % ara) of arabinose. (**g**) Experiments were performed at 30 or 37 °C, as shown. For the imaging data (**a, c, e, g**), graphs show the average proportion of cells with at least one T6SS structure derived from three independent biological replicates (±sd), as indicated by individual dots. (**b, d, f**) Interbacterial killing assays were performed as in Fig. 3. Statistical significance using one-way (**a, b, e, f**) or two-way (**c, d, g**) ANOVA followed by Dunnett’s (**a, e, g**) or Šídák’s (**b–d, f**) multiple comparisons tests is indicated. Significance shown above bars depicts comparisons to A1552 WT, while all other comparisons are indicated. *, *P*<0.05; **, *P*<0.01; ***, *P*<0.001; ****, *P*<0.0001; n.s., not significant.

To better understand what triggered sporadic T6SS production, we re-evaluated regulatory pathways that were reported to influence the T6SS in 7PET strains [38, 39, 56]. By checking diverse mutants, we demonstrated that the OscR regulator, a proposed repressor of the T6SS, had no impact on sporadic T6SS production and, accordingly, low prey killing activity under standard laboratory conditions (Fig. 4a, b). The absence of the VxrAB TCS, on the other hand, significantly reduced the percentage of T6SS-positive cells, a phenotype that was particularly pronounced for the *vxrB* mutant (Fig. 4a), which no longer killed prey under the tested conditions (Fig. 4b). Both phenotypes could be restored to the WT condition by *vxrB* complementation *in cis* (e.g. carried downstream of *P*_BAD_ on a site-specifically integrated miniTn7 construct; Fig. 4c, d). Deletion of the residual genes of the *vxr* operon (e.g. *vxrC, D, E*) did not impact the sporadic T6SS production or interbacterial killing (Fig. 4e, f). Collectively, these experiments suggest that VxrB promotes T6SS production in a small subpopulation of 7PET strains under standard laboratory conditions.

### Sporadic T6SS production increases in the absence of the TsrA repressor

Zheng and colleagues did not observe T6SS activity for the WT strain of the 7PET isolate C6706 (although this strain was later described to be QS-impaired [66], consistent with previously published work on the QS deficiency of certain stocks of strain C6706 [64]). Interestingly, Hcp production and secretion as well as VgrG1-dependent actin crosslinking was detectable after concomitant deletion of *luxO* (encoding a QS repressor) and *VC0070* in this strain background [44]. The *VC0070*-encoded protein was therefore named type VI secretion system regulator A or TsrA [44], which was suggested to act as a transcriptional silencer of virulence and T6SS genes in *V. cholerae *, similar to histone-like nucleoid-structuring protein H-NS [66]. Given this TsrA-dependent T6SS repression under standard laboratory conditions [44], we tested whether this protein contributed to the sporadic T6SS activation in 7PET strains. As shown in Fig. 4(g), this was indeed the case, as a *tsrA* mutant carried significantly more T6SS structures than its parental WT strain. In this mutant, the T6SS structures were also detectable when the bacteria were grown at 37 °C instead of 30 °C (Fig. 4g). Importantly, T6SS production was almost absent in a double mutant that lacked *vxrB* and *tsrA* at both tested temperatures (Fig. 4g). We therefore conclude that the sporadic T6SS activation occurs through VxrB signalling, which is probably enhanced in the absence of TsrA, potentially due to increased DNA accessibility in the absence of this putative nucleoid-structuring protein.

## Conclusion

In this study, we investigated the *V. cholerae* T6SS under standard laboratory conditions. We showed that low levels of T6SS activity are common for 7PET strains and that this activity is based on sporadic production of the machinery in a small fraction of the population. This sporadic activation was dependent on the presence of the QS regulator HapR and the VxrAB TCS. As this TCS is known to be involved in the sensing of cell envelope stress [40], it is tempting to speculate that the *in vitro* growth conditions used in this study might lead to (potentially mild) envelope stress and therefore VxrAB activation within a subfraction of the population. Indeed, phenotypic variability is a frequent feature of clonal populations. Importantly, for several phenotypes, an increased fitness has been demonstrated for heterogenous populations, supporting the idea that bacteria can use bet-hedging strategies to maximize survival [67]. Notably, the observed sporadic (or stochastic) VxrAB-dependent T6SS production phenotype differed significantly from the T6SS status of environmental and non-pandemic *V. cholerae* strains, which contain a deterministic SNP upstream of *vipA* [36, 37]. Indeed, strains carrying this SNP, as well as TfoX-/TfoY-producing 7PET strains, show almost uniform T6SS production within the population consistent with increased T6SS transcript levels compared to those in WT 7PET strains, as demonstrated previously through RNA sequencing (RNA-seq) [24, 28, 36].

Interestingly, we also observed that a temperature of 37 °C lowers the percentage of T6SS-positive cells within the population *in vitro*, which makes us curious about the 7PET strains’ T6SS-dependent phenotype within infected hosts. In this context, Mandlik and colleagues performed RNA-seq analyses using two cholera animal models (infant mice and infant rabbits), whereby most T6SS genes showed no significant expression difference under *in vivo* conditions compared to that in the *in vitro* LB culture condition (table S1 in [68]). However, when tested by quantitative reverse transcription PCR, Fu *et al.* showed that the expression of a subset of T6SS genes was upregulated *in vivo* compared to that *in vitro* [69], although only the transcripts of the two T6SS immunity genes *tsiV1* and *tsiV3* were more than 5-fold enriched in the rabbit-derived samples compared to those *in vitro* [69]. These authors also provided evidence that the T6SS was indeed active *in vivo*, given that the immunity-deficient *tsiV3* mutant was depleted in infant rabbits [69]. Given the large inoculum and ~480 different mutants in each experimental batch, the observed depletion of the *tsiV3* mutant would, however, likewise be expected under standard *in vitro* conditions if contact between the bacteria were promoted. Consistent with this idea, we showed significant prey depletion at such predator-to-prey ratios. Moreover, we also speculate that T6SS production within a small subpopulation would be sufficient to explain the previously demonstrated *in vivo* competition with pre-inoculated T6SS-sensitive *E. coli* prey as well as the observed T6SS-dependent innate immune response in infant mice [26]. The question therefore remains whether human/animal infection conditions, including zebrafish colonization [27], trigger T6SS production beyond the observed *in vitro* levels of 7PET strains, as is the case for natural chitinous surfaces (in a TfoX-dependent manner [22, 24, 28, 29, 36]), or whether no significant induction occurs *in vivo.* If no induction were to occur, we hypothesize that previously demonstrated phenotypes in infant animals reflected subpopulation-based T6SS production similar to the *in vitro* conditions presented in this study. Collectively, such data support our speculation that 7PET strains are SNP-converted to (almost) silence their T6SS under non-inducing conditions, thereby avoiding excess intestinal inflammation [36] and potentially fostering asymptomatic carriage, a common feature of 7PET strains [70].

## Supplementary Data

Supplementary material 1

Supplementary material 2
