# Supplementary material for: Sporadic type VI secretion in seventh pandemic Vibrio cholerae
Source: Microbiology (Reading). 2023 May 3;169(5):001329. doi: 10.1099/mic.0.001329 (PMC10268840; doi:10.1099/mic.0.001329)
Supplement: Supplementary material 1 [file mic-169-1329-s001.pdf]

SUPPLEMENTARY INFORMATION LEGEND

Movie S1: T6SS dynamics in 7PET *V. cholerae*. Strain A1552 carrying the VipA-sfGFP translational fusion was imaged every minute for 30 min. The movies were extracted from the Fiji software after correction of the drift and adjustment of the brightness/contrast of the GFP channel. Three independent replicates were performed.
